# Catalytic pyrolysis of olive oil residue to produce synthesis gas: the effect of bulk and nano metal oxides

**DOI:** 10.55730/1300-0527.3437

**Published:** 2022-05-11

**Authors:** Ebru KARADAĞ, Selva BİLGE, Yusuf Osman DONAR, Ali SINAĞ

**Affiliations:** Department of Chemistry, Ankara University, Ankara, Turkey

**Keywords:** Olive oil residue, catalytic pyrolysis, synthesis gas, nanoparticles, ZnO, MgO

## Abstract

In this study, olive oil residue (OR) biomass was pyrolyzed in the presence of bulk MgO (B-MgO), nano-MgO (N-MgO), bulk ZnO (B-ZnO)), and nano-ZnO (N- ZnO) metal oxides at different temperatures (400, 600, and 800 ºC). Significant results were obtained in terms of synthesis gas formation and CO_2_ reduction. The efficiency distribution of the products obtained as a result of the metal oxide-based pyrolysis process and the effects of metal oxides were examined in detail. Nanometal oxides were synthesized by the hydrothermal method. Characterization of metal oxides was carried out by Brunauer–Emmett–Teller (BET), x-ray powder diffraction (XRD) analysis and scanning-electron microscopy-energy dispersive x-ray (SEM-EDX) techniques. The metal concentration of OR biomass was detected via the x-ray fluorescence (XRF) technique. Tar product properties were evaluated by gas chromatography-mass spectrometry (GC-MS) and Fourier transform-infrared spectroscopy (FT-IR) analyzes. Analysis results show that pyrolytic tar is very similar to diesel and gasoline as it contains significant concentrations of aliphatic and aromatic hydrocarbons in composition. In addition, the composition of noncondensable gaseous products was determined by micro gas chromatography (micro-GC) analysis.

## 1. Introduction

Olive oil production is an important food processing industry for the countries in the Mediterranean region [1]. Respectively, Greece, Italy, and Turkey are considered the greatest oil producers in the World [1]. In the years 2018–2019 ranges of the world’s oil production reached approximately 3.13 million tons [2].

Although olive oil production is very important for these countries, the residue of the olive oil production process causes many problems such as water pollution, soil pollution, and odor at the extraction stage. These environmental problems negatively affect agricultural activities and economic balance. Also, disposal of olive oil residue is a costly process [3,4]. Therefore, it is very important to recycle this waste by an environmentally friendly and cost-effective method.

Nowadays, one of the most widely used thermochemical conversion methods is pyrolysis [5,6]. Various (bio-oil, bio-char, and non condensable gaseous) products are obtained by pyrolysis method and used in various fields [7–9]. Especially, bio-char has a wide application area such as pharmaceutical research, cosmetics industry, adsorbents, soil fertilization, and fuel [10,11]. Bio-oil provides an advantage because it can be used as a valuable and clean fuel with high thermal value with different treatment processes. Noncondensable gases (H_2_, CH_4_, and CO) are used as a source of power and heat [12]. Synthesis gas, which is a mixture of CO and H_2_ gas components, is of particular interest in the industry [13].

Recent publications in the literature consist of catalytic systems to increase synthesis gas composition [13,14]. Some of these are catalytic studies on the conversion of CO_2_ to CO [15]. Al_2_O_3_ [16], MgO [17], SiO_2_ [18], TiO_2_, ZnO [19,20], CeO_2_ [21] and SnO_2_ [22] etc. as the metal oxides are used in catalytic pyrolysis studies. MgO and ZnO metal oxides have some superior advantages. The moderately basic sites in MgO are effective in cleavage of the C– C, C– O, or C– H bonds. Thus, large molecules are transformed into small molecules and increase bio-oil yield [23]. In addition, MgO has very important key features such as accelerating reverse Boudouard (C + CO_2_ ↔ 2CO) and carbon gasification (C + H_2_O↔CO + H_2_ and C + 2H_2_↔CH_4_) reactions, preventing coke accumulation, and contributing to synthesis gas formation [24]. MgO eliminates oxygenated substances in pyrolysis vapor, reducing the formation of CO_2_ and H_2_O [25]. Researchers report that ZnO improves the stability and reduces the viscosity of bio-oil [26]. Especially, ZnO converts CO_2_ into CO by triggering secondary pyrolysis reactions. In the presence of ZnO, more straight-chain alkane derivatives are obtained [19]. Besides all these features, ZnO and MgO have superior properties such as thermal-chemical stability, low-cost and mechanical strength [27].

Metal oxides are called nanoparticles when they reach 1–100 nm size. Nanosized metal oxides have unique chemical, optical, magnetic and electronic properties that differ from their bulk form [28].

Studies on the effects of bulk and nanometal oxide on biomass pyrolysis are available independently in the literature. However, there are only restricted studies in open literature comparing the effects of bulk and nanometal oxides on biomass pyrolysis. The goal of this study is to illuminate the both effect of synthesized nanosized metal oxides and bulk metal oxides on the product distribution of olive oil residue pyrolysis. The other goal of the study is to produce synthesis gas and reducing CO_2_. Already, metal oxide catalysts are much more suitable in terms of synthesis gas production and reduction of water [29]. In addition, the product distribution is interpreted with a mechanistic approach. The surface area differences of metal oxides significantly changed product yield. The results are remarkable in terms of synthesis gas formation.

## 2. Materials and methods

### 2.1 Materials

Zinc-acetate dihydrate [Zn(OAc)_2_.2H_2_O] (Sigma Aldrich), commercial ZnO powder, cetrimonium bromide (CTAB) (Sigma Aldrich) and ethyl alcohol (Sigma Aldrich) were preferred in order to synthesize ZnO nanoparticles. Sodium hydroxide [NaOH] (SigmaAldrich) and magnesium nitrate hexahydrate [Mg(NO_3_)_2_.6H_2_O] (Merck) were used for the synthesis of MgO nanoparticles.

OR, Turkey/Muğla region was supplied from a local olive oil factory. The proximate and ultimate analyses of raw OR was given in Table 1. The metal concentration of OR was showed in Table 2.

It is important that the raw sample contains a low ratio of N and S. Because these compounds cause serious air pollution and decrease the quality of the products formed during the pyrolysis process. Use of olive residue in the pyrolysis process is useful in this regard. Therefore, the pyrolysis of olive waste is advantageous due to its properties. It may be inferred that that it has a very close ash content (3.8% by weight) with other biomass containing about 2% ash by weight, such as wood waste. When Table 1 is examined, it is seen that the carbon content of the raw OR (63.0 wt%) is high. On the other hand, the fixed carbon value in the raw material is very low (29.0 wt%) due to the high volatile matter content (67.2 wt%). Generally, olive residue consists of oil, protein, organic extract, lignin, cellulose, hemicellulose, and holo-cellulose [29,30]. The OR was ground to small sizes prior to pyrolysis and then sieved (0.75 mm). Many researchers have concluded that the optimum particle size for high tar and synthesis yields ranges from 0.60 mm to 1.25 mm. The small particle size is one of the important factors that reduce the synthesis gas yield [30].

Table 2 shows the metal species and concentrations found in the raw OR structure. It is seen that the main metal compounds in the olive oil residue are CaO (7.07 wt%) and K_2_O (3.47 wt%). These results show that the char product obtained from olive oil residue can be used in soil applications.

The tar composition distribution obtained as a result of the pyrolysis process performed without catalyst at different temperatures is shown in Table 3. According to the results, the increase in the pyrolysis temperature increased the amount of carbon in the tar composition. The highest carbon content is observed at 800 ºC.

### 2.2. Methods

#### 2.2.1. Synthesis of B-MgO

B-MgO was synthesized according to the studies in the literature [31]. B-MgO was synthesized by a conventional technique. In this technique, Mg(OH)_2_ was first dissolved in double-distilled water. Then NH_3_ was added slowly to the mixture. After this step, the mixture was precipitated at room temperature for 24 h. The particles were recovered by centrifugation and washed.

#### 2.2.2. Synthesis of B- ZnO

B-ZnO was synthesized according to the studies in the literature [32]. Powdered ZnO was calcined in a muffle furnace at 800 ºC for 2 h. The powder cooled at room temperature was crushed in a mortar. In order to remove the impurities in the sample, it was washed 2 times with distilled water and dried in an oven at 150 ºC for 1 h.

#### 2.2.3. Synthesis of N-ZnO nanoparticles

In the first step, 30 mL of ethyl alcohol and 2.5 g of Zn (OAc)_2_.2H_2_O were mixed rapidly for 15 min. In the second step, 0.5 g surfactant CTAB was added to the homogeneous mixture and after mixing (4000 rpm) for a while it was put into the autoclave. The mixture was reacted at 140 ºC for 6 h. The highest pressure value observed during the reaction was 4 bar. The product obtained was washed with a mixture of ethyl alcohol and double distilled water (1:1) until the pH was neutral. Finally, the neutralized product was dried at 120 ºC for 2 h.

#### 2.2.4. Synthesis of N-MgO nanoparticles

As starting materials, Mg (NO_3_) _2_.6H_2_O and NaOH were used for synthesis. An equal amount of distilled water and ethyl alcohol was used as the solvent during the experiment. A total of 4 mg NaOH was mixed in a 40 mL solvent mixture until homogeneous. Approximately 2 g of Mg (NO_3_) _2_.6H_2_O was transferred to the prepared solution and stirred. The obtained white mixture was placed in a 75-mL autoclave. The mixture was reacted at 130 ºC and 14 h. The product obtained was washed with a mixture of ethyl alcohol and double distilled water (1:1) until the pH was neutral. Finally, the neutralized product was dried at 120 ºC for 2 h.

#### 2.2.5. Pyrolysis process procedure

The pyrolysis process was performed at 400, 600, and 800 ºC with the system shown in the previous study [20]. Experiments of pyrolysis were performed out at a 20 °C min^−1^ heating rate and for an hour. A total of 3 g OR and 2 % (w/w) catalysts were prepared by hand-mixing and then placed into the furnace. Before the heating, whole pyrolysis system was purged by nitrogen gas about 15 min with a flow rate of 30 mL min^−1^. A quartz reactor in the tubular furnace was used during the experiments. Then, the quartz reactor outlet was connected to the cooling unit and the cooling unit to the gas collection bag. The spiral cooling unit was placed into a Dewar vessel. Dewar vessel was filled with ice-ethyl alcohol mixture and the temperature was kept within the range of -8 ± 2 ºC. Prevacuumed tedlar-bag was used as the gas-collecting unit. In this way, the gas-vapor mixture coming from the reactor was condensed in the spiral cooling unit with ethyl alcohol-ice mixture, and the liquid product was collected by condensing the vapor, and the gas product was passed to the tedlar-bag. In this study, the liquid and solid products were called as tar and char, respectively. Each experiment was repeated 3 times and optimum results of each repeat series were used.

#### 2.2.6. Analytical procedure

Gas components were determined by SRA Instruments (T3000 model micro GC) device. Equations related to the yields of the products were mentioned in this study [31]. The structure of the samples was determined by means of FT-IR Thermo Scientific Nicolet- iS10 analysis. The IR spectrum was taken by distributing the samples on the KBR pellet. The tar composition obtained was determined by GC-MS (AGILENT-6890-5973- MSD). HP1 (50 m × 0.32 mm × 0.52 m) type column was used for GC-MS analysis. Also, helium (flow rate: 0.7 mL min^−1^) was used as the carrier gas, and it was studied at the specified temperatures (temperature ramp: 300 ºC at 5 ºC min^−1^). Compounds found in the obtained tar samples were indicated as the peak area (%) on the total chromatogram, compared with the data from the NIST library. The surface morphology of bulk and nanometal oxide samples were illuminated by SEM- EDX analysis. In addition, the crystal structure of these materials was examined by XRD analysis technique. The surface areas of metal oxide samples were measured by BET technique. The metal concentration of OR biomass was detected via the XRF technique. The elemental composition of the raw olive oil residue was carried out in the LECO- 932- CHNS elemental device. Temperature programmed desorption of NH_3_ (NH_3_-TPD) was applied to characterize the acidic properties of catalysts by using Micromeritics ChemiSorb 2720 instrument. A total of 30 mg of powder samples were loaded in a quartz tube, placed into the furnace and heated to 220 ºC under helium flow (15 mL min^−1^) to remove possible impurities remain on the samples. Then, the temperature was decreased to 80 °C and the system was introduced with NH_3_ (15 mL min^−1^flow rate) for 30 min. Then, the system was purged by using helium gas with 25 mL min^−1^ flow rate for the removal of any possible NH_3_ remaining. Finally, catalysts were heated from 35 ºC to 750 ºC under helium flow at a heating rate of 10 ºC mL min^−1^and ammonia desorption was monitored by TCD.

## 3. Results and discussion

### 3.1. Material characterization

Desorption of ammonia at high temperatures (higher than 450 ºC) corresponds to strong acid sites. Desorbed ammonia between 100 and 250 ºC indicates the presence of weak acidic sites. For medium acid sites, desorption temperature is in the range of 250–450 ºC [33]. It can be seen from Figure SI1 that both N-MgO and B-MgO are in the same temperature range of desorption. N-MgO has a sharper and broad peak compare to B-MgO. Desorption temperature starts from approximately 250 ºC and ends at 700 ºC. This range means that both N-MgO and B-MgO have mainly medium acid sites. But N-MgO showed the highest acidic strength among synthesized catalysts. On the other hand, N-ZnO showed the lowest acidic strength and B-ZnO has a relatively higher acidity than that of N-ZnO (Table 4). The acidity of a catalyst has an important effect on bio-oil upgrading process since it can catalyze deoxygenation, decarboxylation, isomerization and aromatization reactions [34]. In this context, acidity of N- MgO has the highest acidity among synthesized catalyst. Therefore, it can be said that maxium total conversion ratio could be related to N-MgO acidic strength.

**Figure 1:**
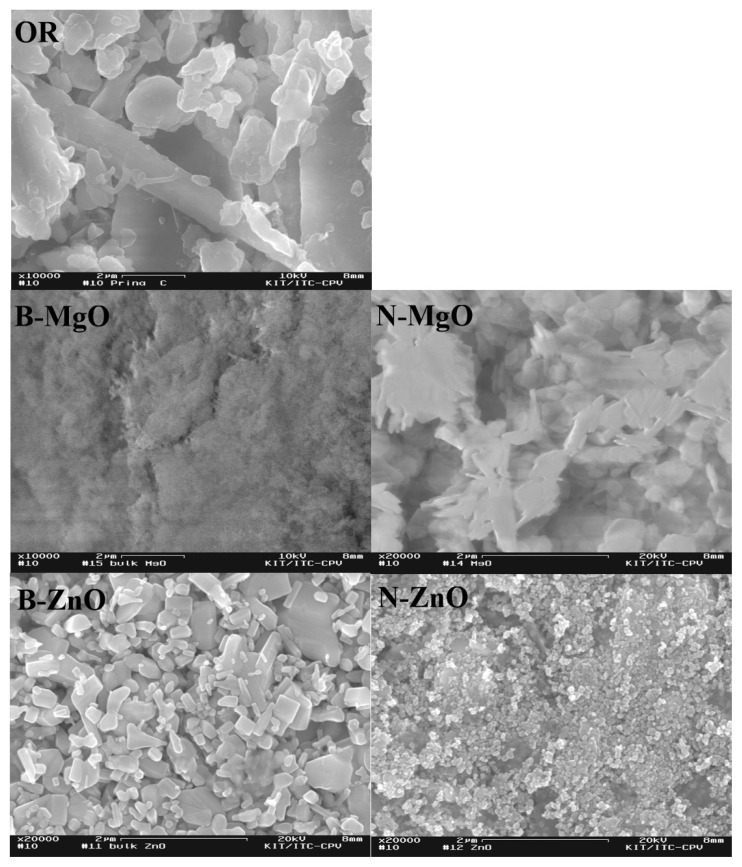
SEM micrographs of the olive residue and metal oxides.

Figure 1 shows surface images of OR and metal oxide samples. EDX results of all samples are available in the supplementary file (Figures SI2-SI4). When the surface images are examined, it is seen that the particle shapes of B-MgO is irregular because of agglomeration. Results for N-ZnO and B-ZnO show that the particles do not have agglomeration problems and are distributed in a regular, controlled manner. No agglomeration occurred due to the use of a surfactant during the synthesis stage. In addition, B-ZnO is in the tubular form in images, while N-ZnO is observed in the spherical form.

The surface areas of metal oxide samples are shown in Table 5. The results show that there are considerable differences between bulk and nanomaterials in terms of surface area size. N-ZnO and N-MgO have the highest surface area. High surface area metal oxides have some advantages since they contain large amounts of active sites. These active sites contribute to the realization of secondary pyrolysis reactions and increase the gaseous product efficiency [19].

Figure SI5 provides information on the crystallite size of the bulk and nanoparticles. When the diffraction patterns of MgO nanoparticles are examined, peaks (1 1 1), (2 0 0), and (2 2 0), are seen at different angles, 38.0º, 43.0º, and 61.1º, respectively. Also, the peak at 38. 0º is an indication of the presence of the Mg(OH)_2_ phase.

The crystallite size of the synthesized nanoparticles, Dc, is found from the main (111) diffraction peaks using the Scherrer equation;


$$Dc=\frac{K\lambda }{\beta cos\theta }.$$

In this formula, “θ” is the Bragg angle, “K” is the constant value, “λ” is the x-ray wavelength used in the analysis, and “β” is the pure diffraction broadening of a half-height peak. The diameter of the nanoparticles was calculated as 10.9 nm using the Scherrer equation.

It was determined that the diffraction patterns for the ZnO nanoparticles (Figure SI5) correspond exactly to the JCPDS file No. 36- 1451. In addition, it was proved that the major peaks of ZnO nanoparticles correspond to zincites with hexagonal structure [28].

### 3.2. Pyrolysis product yields

**Figure 2:**
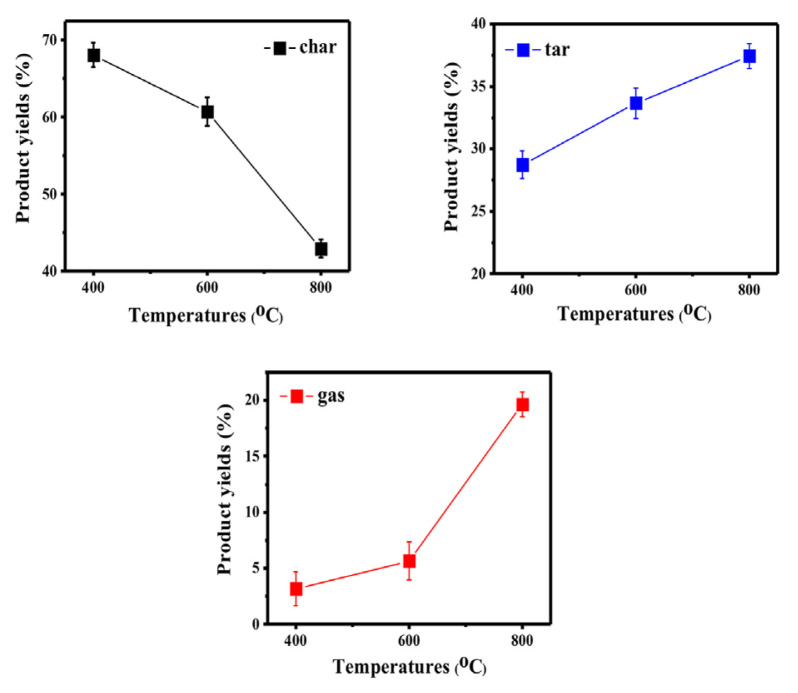
Effect of different temperatures on the yield of products without catalyst.

In Figure 2, the distribution of product yields obtained from olive waste at 400, 600, and 800 ºC is given. When the results are examined, it is seen that as the pyrolysis temperature increases, char yield decreases due to char elimination reactions [22,35,36], and tar and gaseous product yield increases. In particular, a significant increase in gaseous product yield is observed at 800 ºC. The conversion of char to gas products occurs as a result of some possible reactions on the carbon surface [37] such as these reactions,

Reaction-1 C + CO_2_ ↔ 2CO
Reaction-2 C + H_2_O ↔ CO +H_2_
Reaction-3 C + 2H_2_ ↔ CH_4_
Reaction-4 CH_4_ + H_2_O ↔ CO +3H_2_.

The interpretation of the findings related to the distribution of gaseous products and product yield is based on reactions given above. These reactions are referenced when evaluating the effect of different metal oxide catalysts on the composition of gaseous products.

### 3.3. Effect of different metal oxides on product yields

**Figure 3:**
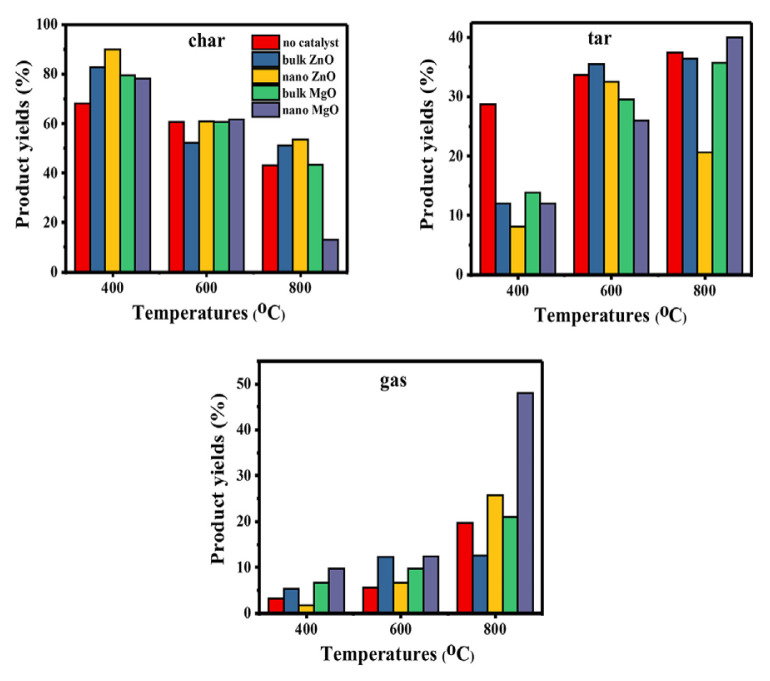
Effect of different metal oxides on product yields at different temperatures.

**Figure 4:**
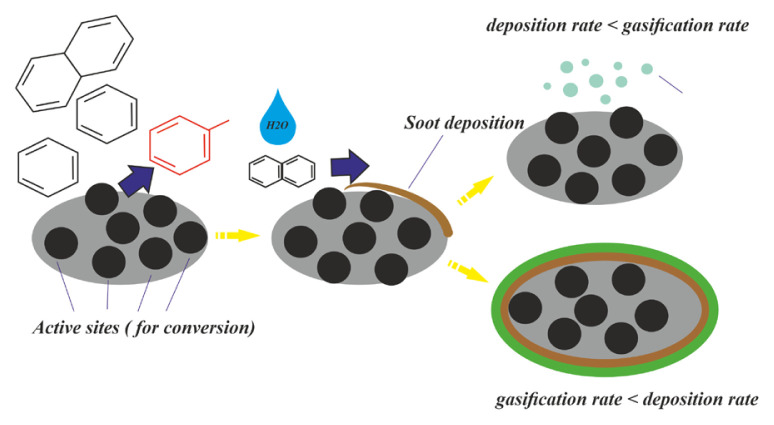
Conversion mechanism of products.

Figure 3 shows the product distribution as a result of the catalytic pyrolysis process carried out under different temperatures. At 400 ºC, all catalysts blocked the char elimination reaction and reduced the yield of tar and gaseous products. This is due to the adsorption of tar in active sites on the surface of the char formed during the catalytic reaction. It can also occur in some polymerization reactions. Thus, a second surface occurs consisting of hydrogen and soot (Figure 4). This soot blocks the active sites, hindering the interaction of the active sites with the gaseous tar [38].

By the increase of temperature, nanometal oxides increased gaseous product yield significantly. Especially, N-MgO significantly increased gaseous product yield by performing the reaction 1. Detailed gas analysis findings also support this result.

In some cases, an increase in the pyrolysis temperature at relatively low temperatures (< 600 ºC) facilitates the evaporation of organic compounds and therefore increases the porosity of the char formed. However, at higher temperatures (600 ºC >), a significant increase in temperature causes more ash formation. These pores can become blocked, resulting in shrinkage of micro/macropores and a reduction in open porosity [38]. For this reason, especially for B-ZnO, it may have performed poorly in terms of gas product yield at high temperatures.

### 3.4. Effect of different metal oxides on total conversion ratio

**Figure 5:**
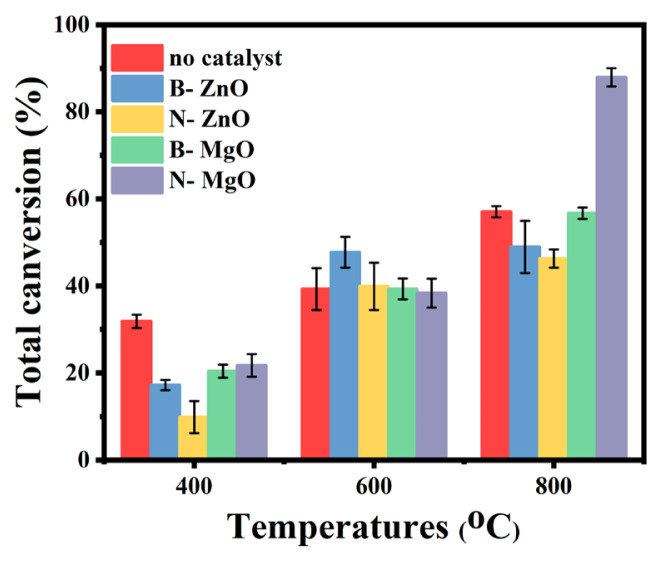
Effect of metal oxide catalyst on total conversion ratio.

In order to better determine the effect of bulk and nanometal oxides on tar and gaseous products, the total conversion rates are given in Figure 5. The total conversion rate consists of the amount of tar + gaseous product yields. N-MgO realized the highest conversion rate (87.94 %) at 800 ºC. The gaseous product formed at high temperature clearly affected the total conversion rate. In addition, B- ZnO showed a better conversion rate (47.75%) than no catalyst results at 600 ºC. However, when the temperature increased, the yield of gaseous and tar products decreased with the effect of B-ZnO, which decreased the conversion rate.

### 3.5. Effect of different metal oxides on gaseous products distribution

**Figure 6:**
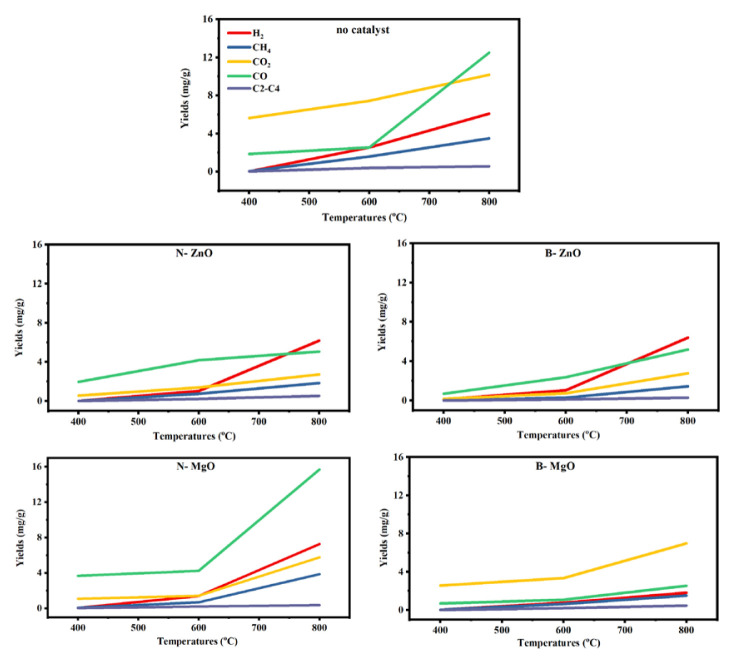
Effect of different metal oxides on the distribution of gaseous products at different temperatures.

Figure 6 shows the composition of gaseous products formed as a result of catalytic reactions. The results indicate that bulk and nanometal oxide samples catalyzed reaction 1 and significantly reduce the amount of CO_2_. On the other hand, bulk and nano-ZnO may catalyzed the reaction 2 because the amount of H_2_ and CO were increased significantly. Also reaction 4 may have occurred due to the slight decrease in the amount of CH_4_. These catalysts produced the highest synthesis gas composition (H_2_ + CO) at 800 ºC. The results are a successful approach to synthesis gas production on a laboratory scale. N-MgO increased the amount of CO about eight times more than B-MgO. In addition, the N-MgO catalyst reduced the amount of CO_2_ more than all of them. Some researchers support that MgO is effective in carbon gasification reactions [23,39,40]. However, in this study, the effect of nanosized MgO has been proven to be better than that of bulk MgO. Because N-MgO has a higher surface area and contains a number of basic regions. These regions play a role in the elimination of oxygenated compounds as noncondensable gas products such as CO [41,42]. Use of basic MgO can significantly increase the resistance to carbon formation [41].

Catalysts are divided into three classes: biological, homogeneous and heterogeneous [42]. Among all these, the role of heterogeneous catalyst in pyrolysis applications is critical in terms of its resistance to conditions such as high temperature and pressure, its stability and its easy recovery from products. For this reason, nanometal oxides used in pyrolysis applications are of great importance due to the active sites they contain [43]. The heterogeneous catalyst properties of nanometal oxides are due to the reactivity of the active sites, particle size and different morphologies. However, strong binding of the nanosized heterogeneous catalyst to the surface, for example, the defect sites on the surface or the functional groups attached to the surface, and the catalyst itself is required [44]. At this point, the agglomeration parameter plays a critical role. Because agglomeration reduces the effectiveness of active sites where gas conversion reactions ocur [44]. In this context, establishing the synthesis procedure of nanoparticles is decisive. In this study, agglomeration was prevented by using surfactant while synthesizing ZnO nanoparticles. In addition, when the SEM images for N- ZnO and N-MgO are examined, it can be seen that the material morphologies can be observed clearly.

### 3.6. Effect of different metal oxides on tar composition

**Figure 7:**
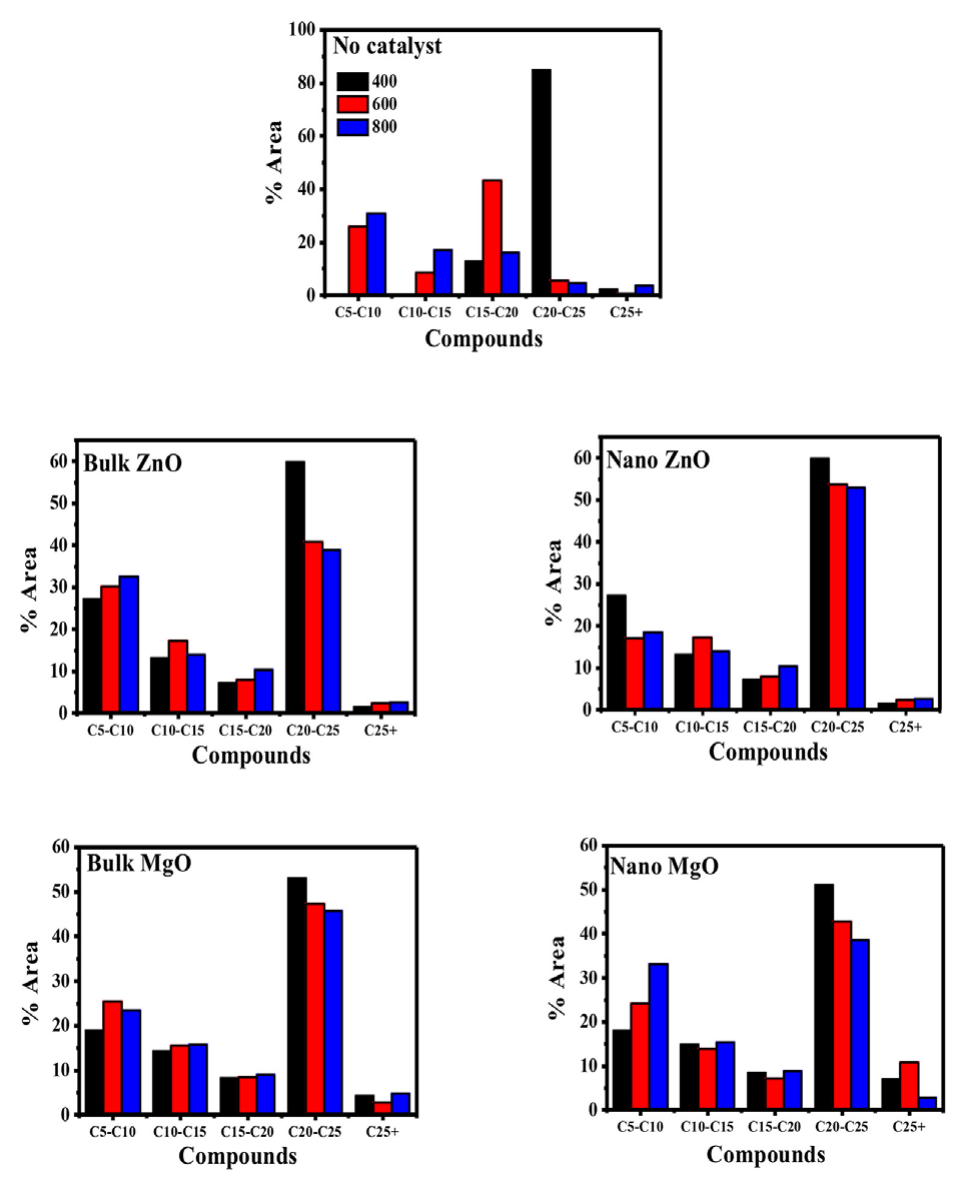
Effect of different metal oxides on the distribution of tar composition.

Figure7 shows the tar product composition resulting from the catalytic pyrolysis process. Experimental results without catalyst at 400 ºC show that the tar composition mostly consists of C20-C25 groups. It is seen that when the temperature increases, these groups decrease. The mesoporous and macroporous MgO catalyst with strong cracking characteristics is used for biomass pyrolysis and gasification for the cracking of large molecules [25]. In the presence of B-MgO, the C5–C10 groups increase compared to the results without the catalyst. This raise reaches the highest level in the presence of N-ZnO at 800 ºC.

Catalysts, effectively promote the cleavage of the C–H, C–O, or C–C bond and also promote the cracking of large molecules to small molecules, thereby improving the yield of tar selectively [23].

Consequently, GC-MS analysis of catalyzed pyrolytic tar indicates the pyrolytic tar is partially similar to diesel (C8–C24) than gasoline (C5–C12) with their significant concentrations of aliphatic and aromatic hydrocarbons.

FT-IR spectra table are given in the supplementary file (Figures SI6–SI8, and Table SI1). FT-IR results support that high molecular weight structures turn into small and light molecules in the presence of catalysts. Aromatic C-H bond stretching between 3100 and 3000 cm^−1^ gives information about the hydrogens of aromatic structure [43]. The C-H bond stretching at 3000–2840 cm^−1^ refers to straight-chain alkanes [44]. C=O stretching vibrations with absorbance between 1770 and 1700 cm^−1^ also indicate the presence of ketones and aldehydes in tar [45]. C=O bond stretching in the range of 1600– 1580 cm^−1^ shows the olefinic alkene derivatives [46]. Bond vibrations in the range of 1550–1500 cm^−1^ can be caused by nitro groups [47]. O- H bond vibrations between 1350 and 1260 cm^−1^ indicate the presence of phenols and alcohols [48,49].

## 4. Conclusion

When the results of the experiment and analysis were evaluated, some important determinations were made.

- Nano-ZnO and MgO have a much higher surface area than bulk form. This situation has significantly affected product yields and types.
- N-MgO caused the highest synthesis gas (CO+H_2_) formation. While making this determination, basic character and surface area of N-MgO were taken into consideration.
- In addition, N- MgO achieved the best total conversion ratio. N-MgO increased the efficiency of the product by triggering char elimination reactions.
- B-ZnO increased tar yield at 600 ºC and this result affected the total conversion ratio.
-All of the bulk and nanometal oxides significantly reduced CO_2_ emissions.
-Heavy tar fractions were eliminated thanks to catalysts.
-The differences between bulk and nanomaterials were evaluated with analysis results and a mechanistic approach.

## Supplementary materials

Figure SI1 NH_3_-TPD profiles of synthesized catalysts.

Figure SI2 EDX analysis result of olive residue.

Figure SI3 EDX analysis result of B-ZnO and N-ZnO.

Figure SI4 EDX analysis result of B-MgO and N-MgO.

Figure SI5 Crystallite size of OR and metal oxides.

Figure SI6 FTIR spectrums of the pyrolytic tar at 400 °C.

Figure SI7 FTIR spectrums of the pyrolytic tar at 600 °C.

Figure SI8 FTIR spectrums of the pyrolytic tar at 800 °C.

**Table SI1:** Evaluation of FTIR spectra of tar.

*Wavelength (cm* *^−1^* *)*	*No catalyst*	*B-ZnO*	*N-ZnO*	*B-MgO*	*N-MgO*
*400 °C*					
*3100–3000*	++	++++++	−	++	++
*3000–2840*	++++	+++++	++	+++	+++
*1770–1700*	++	+++	+	++	++
*1600–1580*	−	++	−	+	−
*1550–1500*	−	−	−	+	−
*1470–1350*	+	++	+	+	+
*1350–1260*	−	−	+	++	−
*1342–1266*	+++	++++++	−	++	++
*600 °C*					
*3100–3000*	++++++	++++	++	+++++	+++
*3000–2840*	++	+	+++	++	+
*1770–1700*	++	++	++	+	++
*1600–1580*	−	++	−	−	−
*1550–1500*	−	−	−	+	−
*1470–1350*	−	−	+	+	+
*1350–1260*	−	−	−	−	−
*1342–1266*	++++	+++	+++	++	++
*800 °C*					
*3100–3000*	+++++++	++++	−	++	++
*3000–2840*	++++	++	++	++++	+++
*1770–1700*	+++	++	++	++	++
*1600–1580*	+	++	−	−	+
*1550–1500*	−	++	−	−	++
*1470–1350*	+	++	+	+	++
*1350–1260*	++	−	−	−	−
*1342–1266*	++++	+++	++	+++	+++

## Figures and Tables

**Table 1:** Proximate and ultimate analyses of raw OR.

*Proximate analysis (wt%, dry)*				*Ultimate analysis (wt%, dry)*				
Moisture	Ash	Volatile compounds	Fixed carbon	C	H	N	S	O a
8.0	3.8	67.2	29.0	63.0	4.5	1.7	1.2	29.6

a oxygen amount calculated from difference.

**Table 2:** Metal concentration of raw OR (wt%).

*CaO*	*K* *_2_* *O*	*Si* *_2_* *O*	*SO* *_3_*	*Fe* *_2_* *O* *_3_*	*P* *_2_* *O* *_5_*	*MgO*	*Al* *_2_* *O* *_3_*
7.07	3.47	1.81	0.96	0.64	0.62	0.39	0.37

**Table 3:** Elemental composition of tar OR (without catalyst).

Element (wt%)	400 °C	600 °C	800 °C
**C**	69.25	74.38	76.20
**H**	9.04	9.50	9.69
**N**	1.30	1.72	1.76
**S**	0.15	0.21	0.19
**O** *	20.26	14.19	12.16

* oxygen amount calculated from difference.

**Table 4:** Acidic strengths of synthesized catalysts.

*Catalyst*	*Acidic strength (mmol NH* *_3_* */g catalyst)*
B-MgO	1.09
N-MgO	2.82
B-ZnO	0.15
N-ZnO	0.04

**Table 5:** BET surface areas of the metal oxides.

Metal oxides	Surface areas (*m^2^ g^−1^)*

*B-ZnO*	5.4
*N-ZnO*	208.0
*B-MgO*	107.0

*N-MgO*	232.2
