# Supplementary figures and images for: Catalytic pyrolysis of olive oil residue to produce synthesis gas: the effect of bulk and nano metal oxides
Source: Turk J Chem. 2022 May 11;46(4):1306–15. doi: 10.55730/1300-0527.3437 (PMC10395787; doi:10.55730/1300-0527.3437)

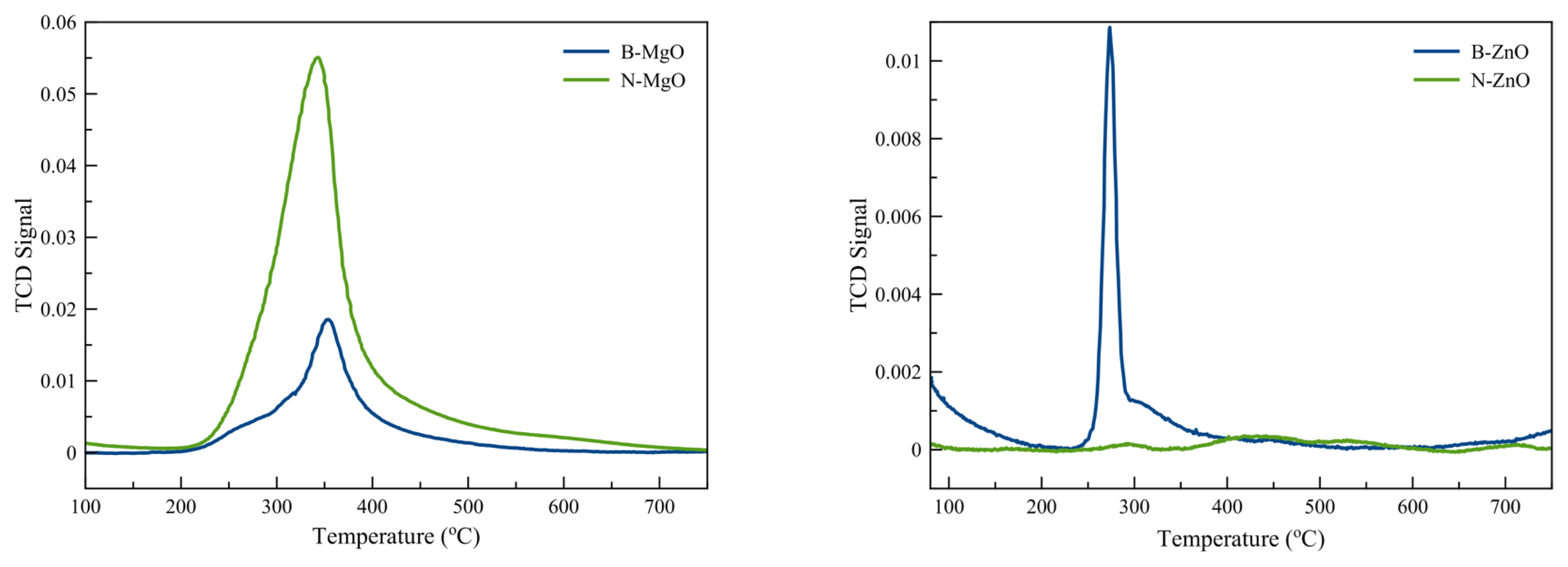

Supplement: Figure SI1 — NH3-TPD profiles of synthesized catalysts. [file turkjchem-46-4-1306s1.tif]

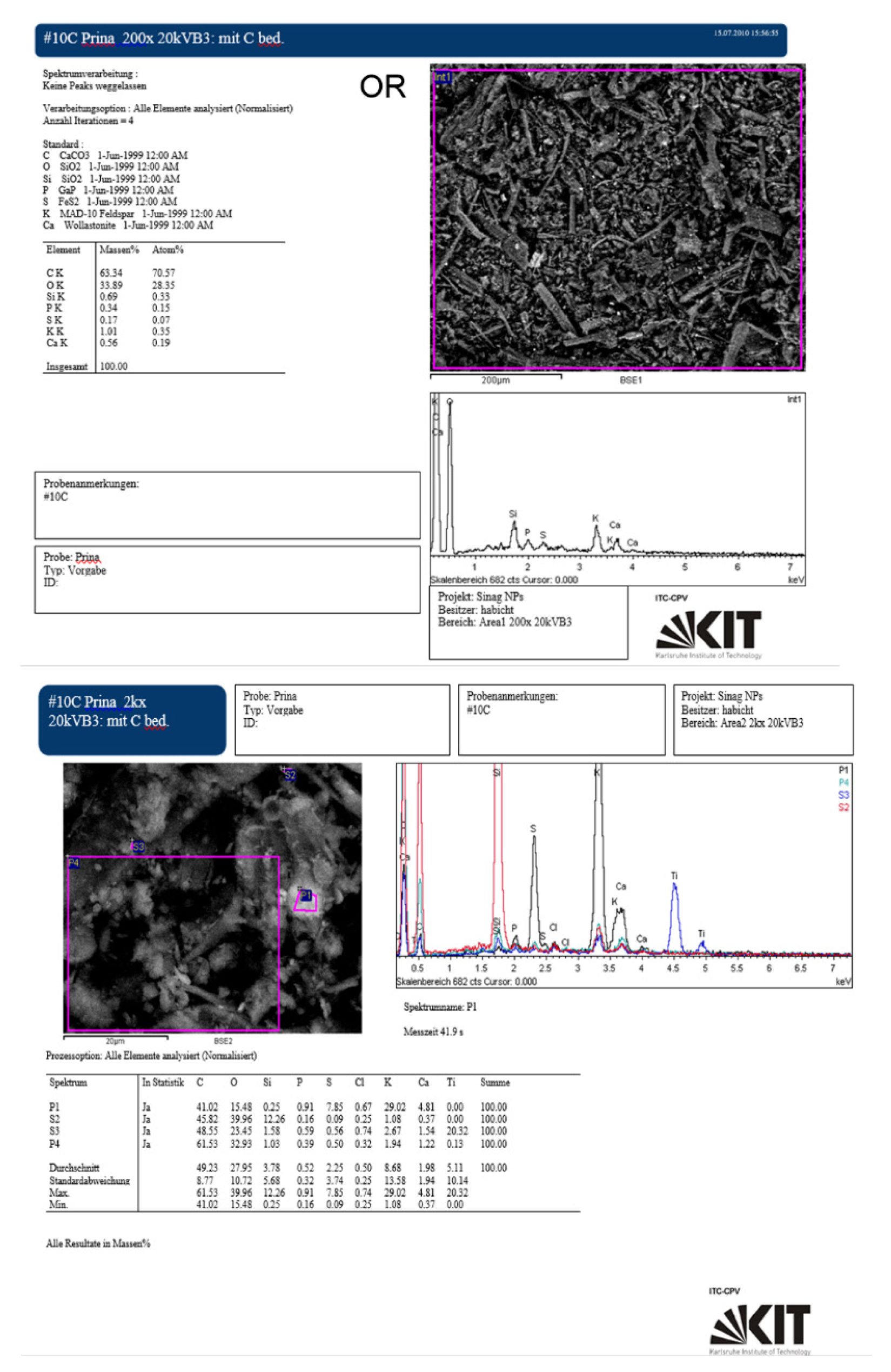

Supplement: Figure SI2 — EDX analysis result of olive residue. [file turkjchem-46-4-1306s2.tif]

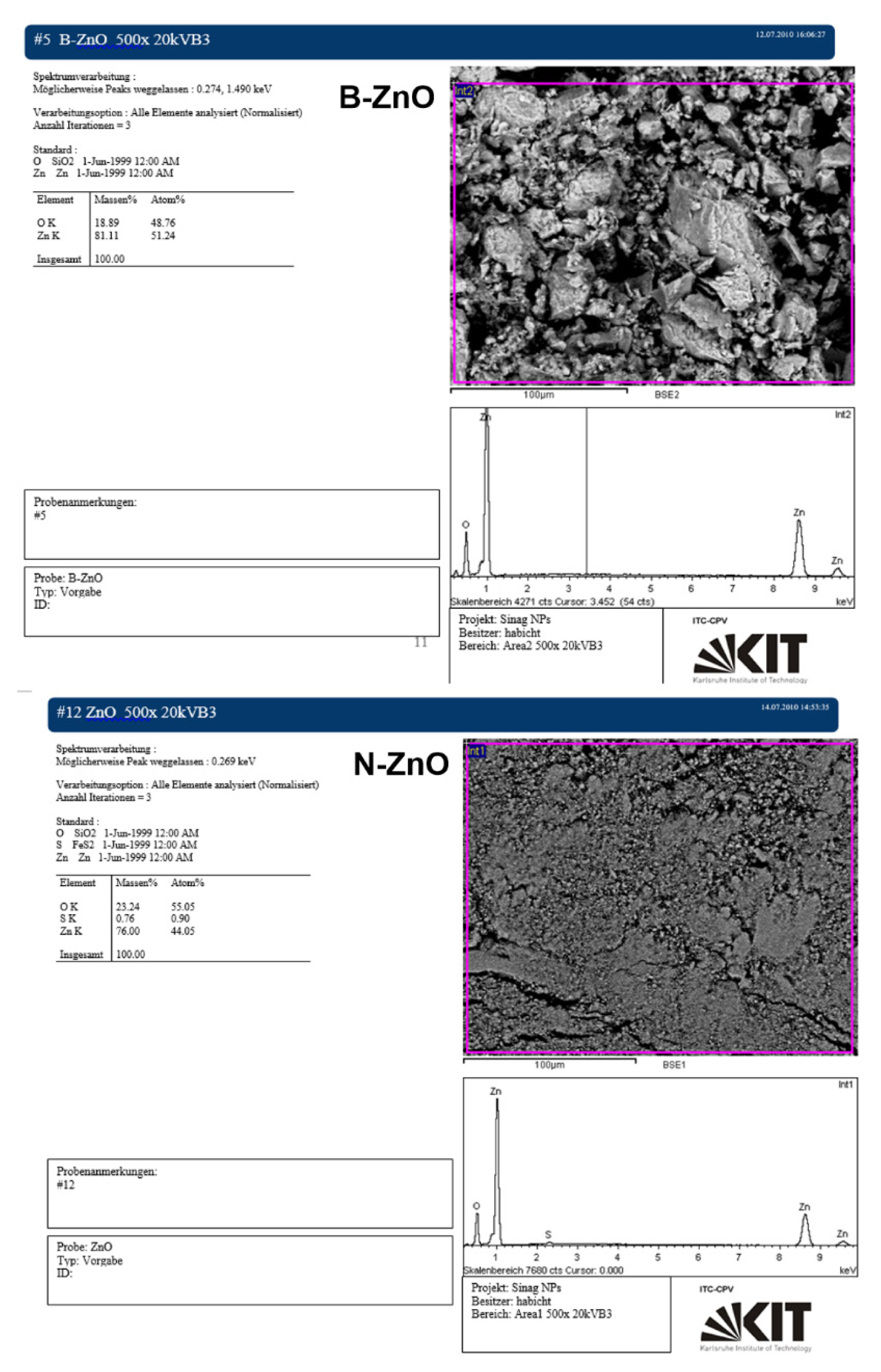

Supplement: Figure SI3 — EDX analysis result of B-ZnO and N-ZnO. [file turkjchem-46-4-1306s3.tif]

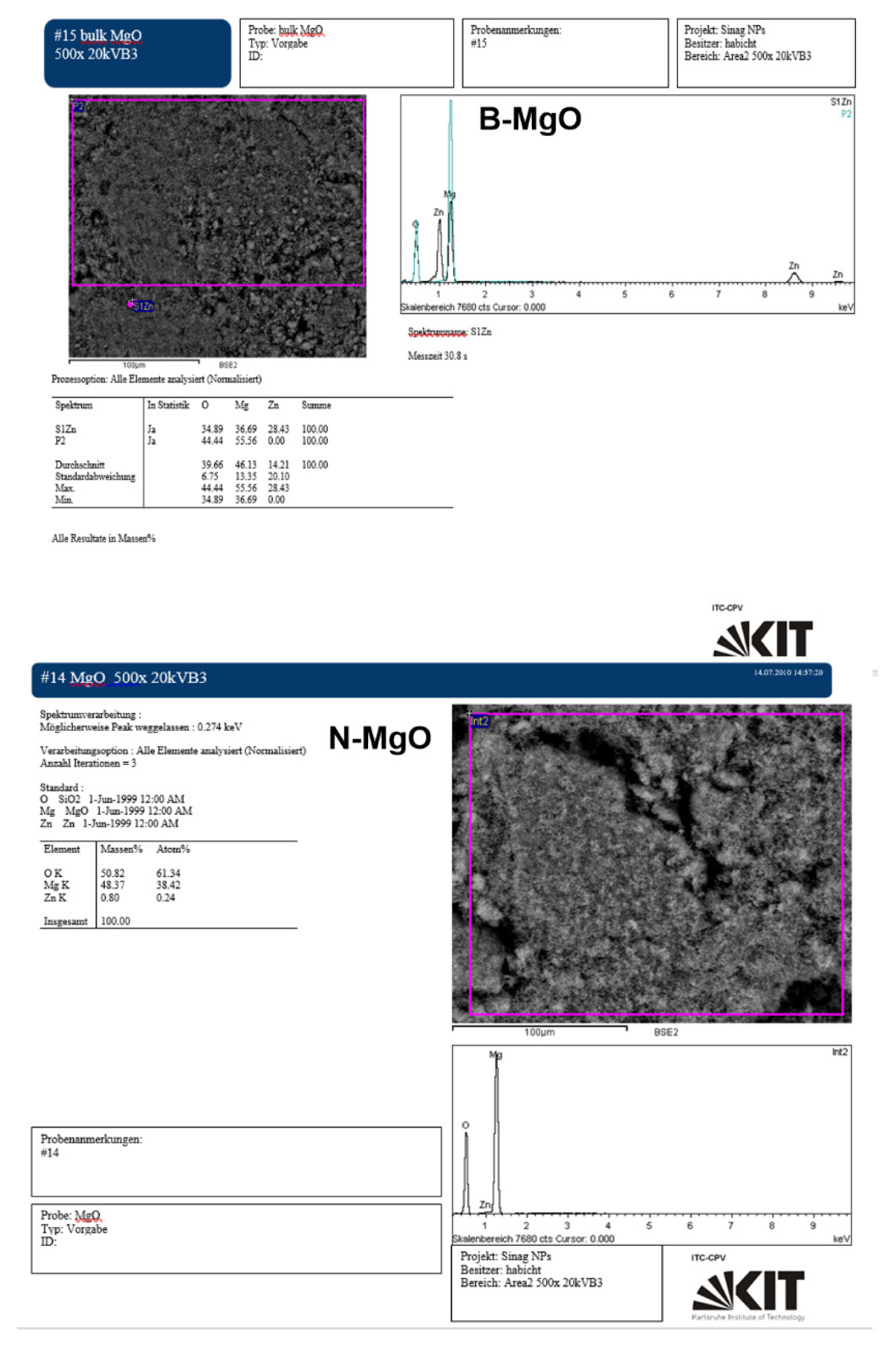

Supplement: Figure SI4 — EDX analysis result of B-MgO and N-MgO. [file turkjchem-46-4-1306s4.tif]

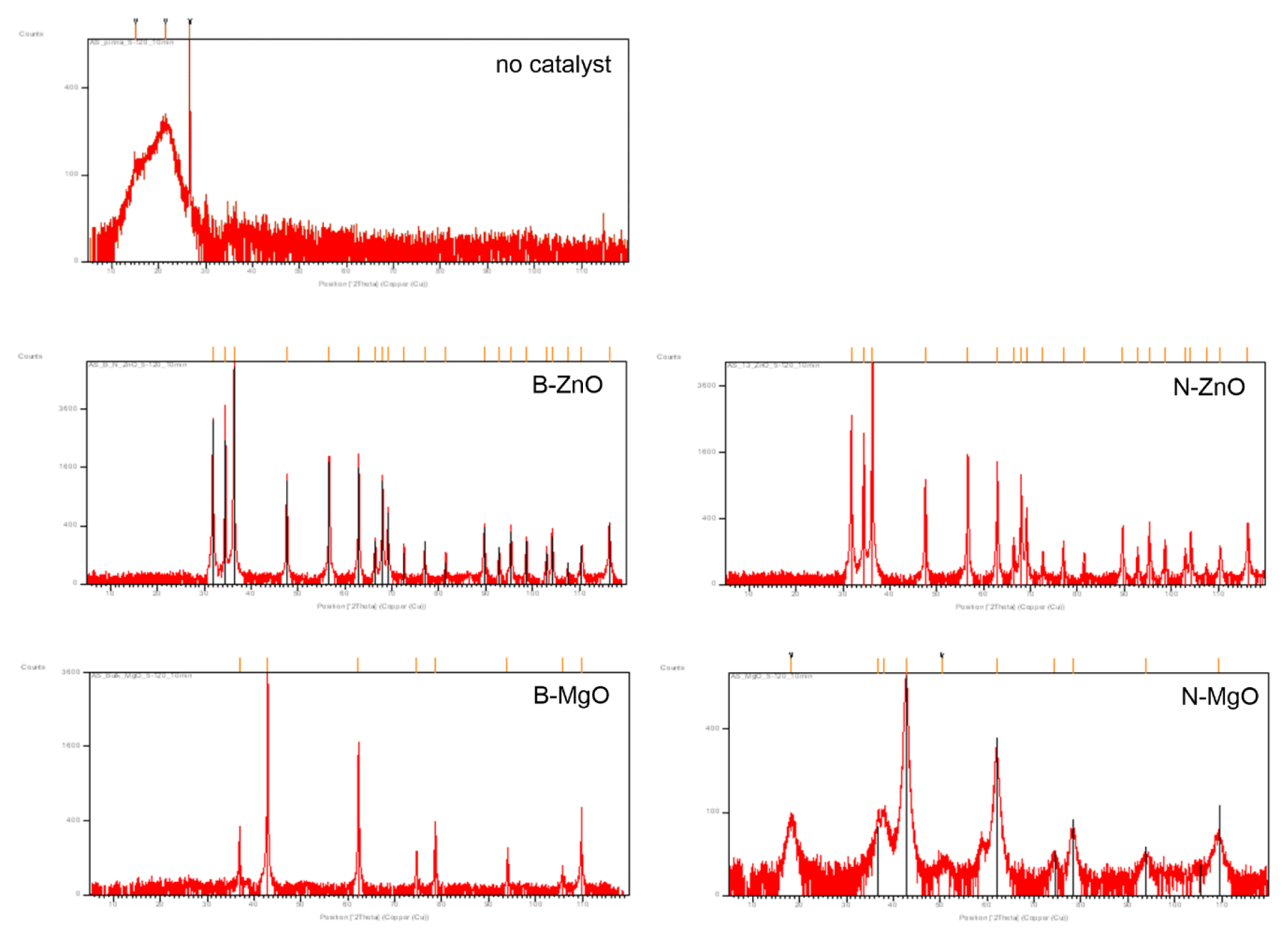

Supplement: Figure SI5 — Crystallite size of OR and metal oxides. [file turkjchem-46-4-1306s5.tif]

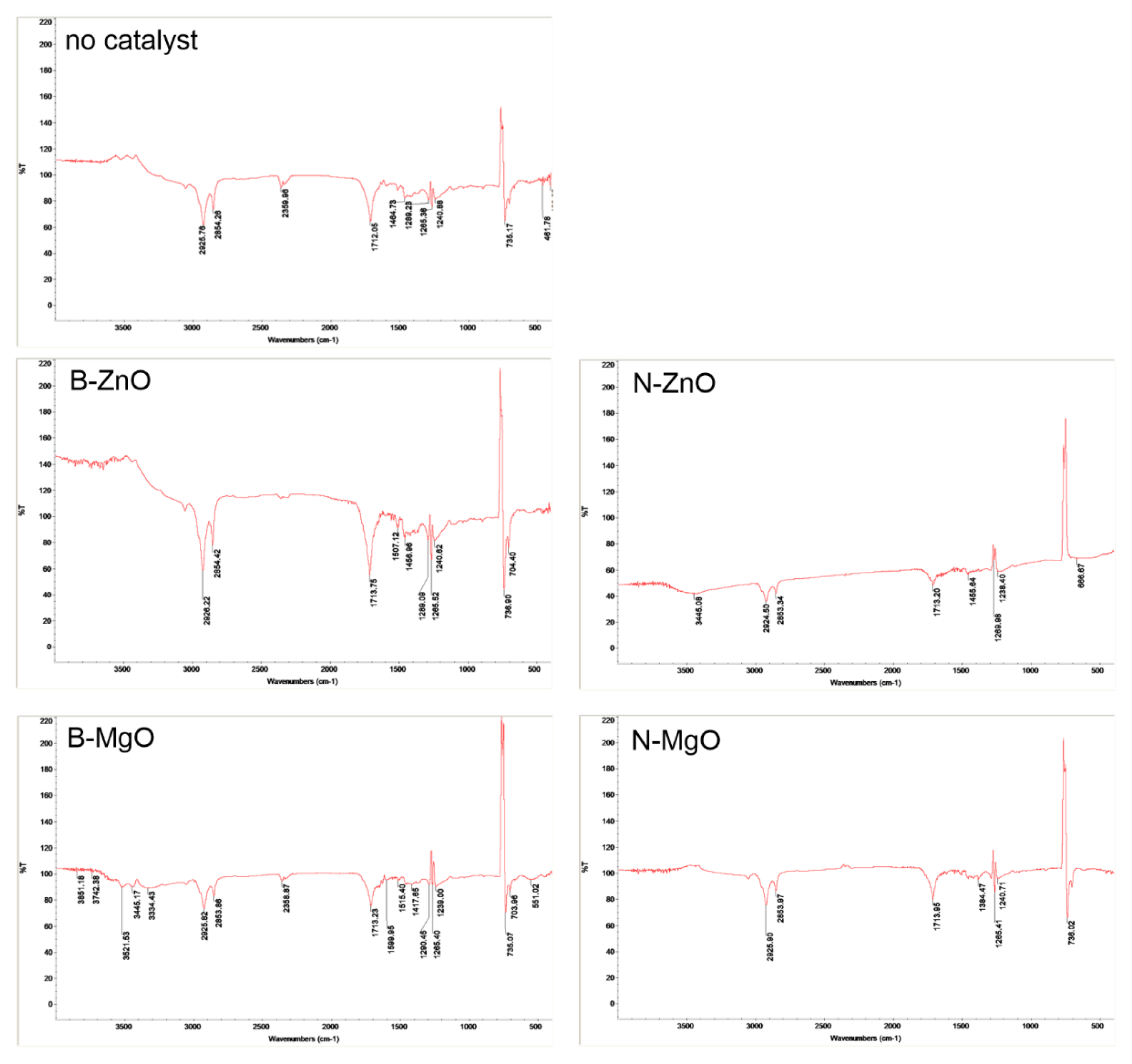

Supplement: Figure SI6 — FTIR spectrums of the pyrolytic tar at 400 °C. [file turkjchem-46-4-1306s6.tif]

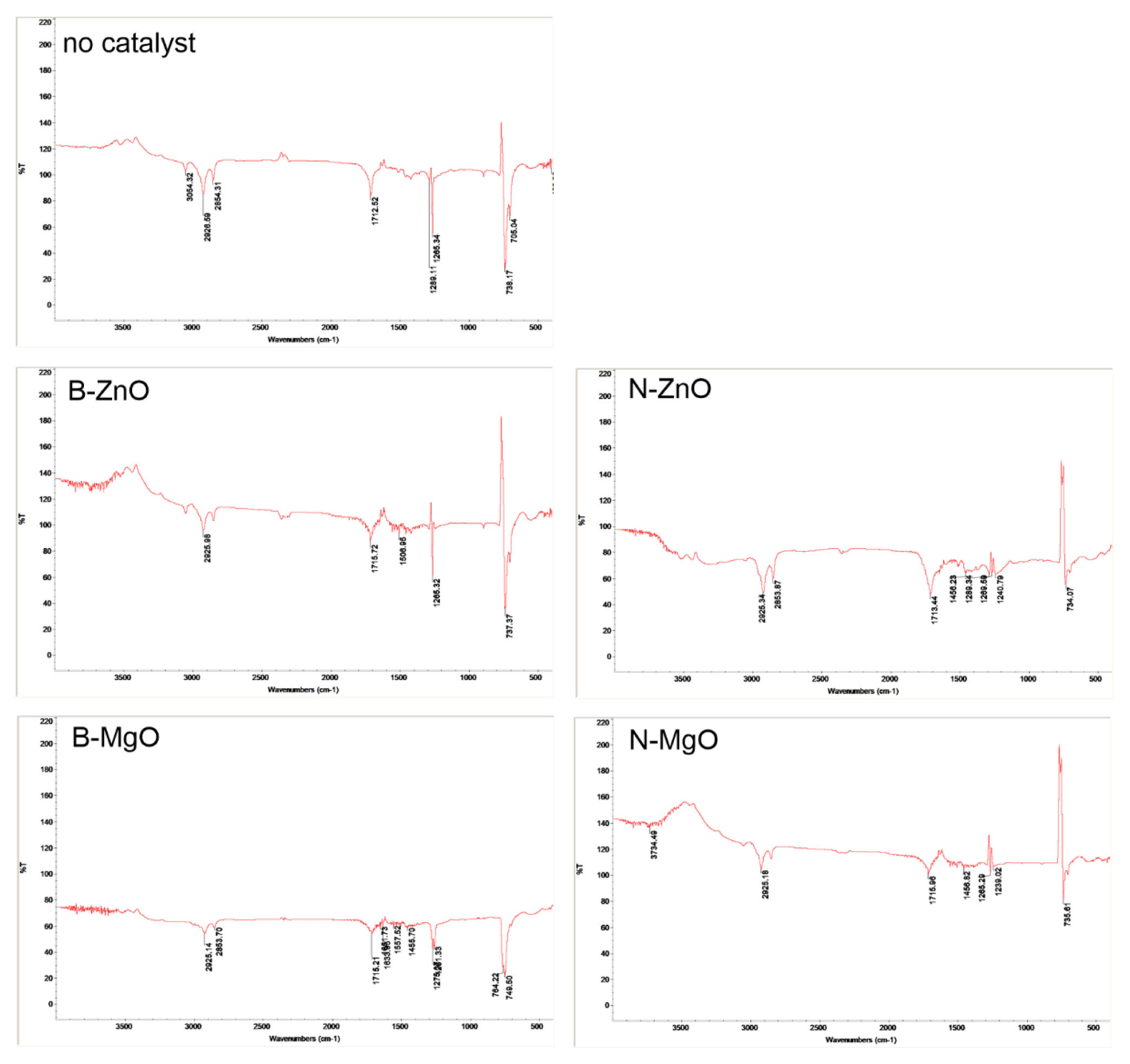

Supplement: Figure SI7 — FTIR spectrums of the pyrolytic tar at 600 °C. [file turkjchem-46-4-1306s7.tif]

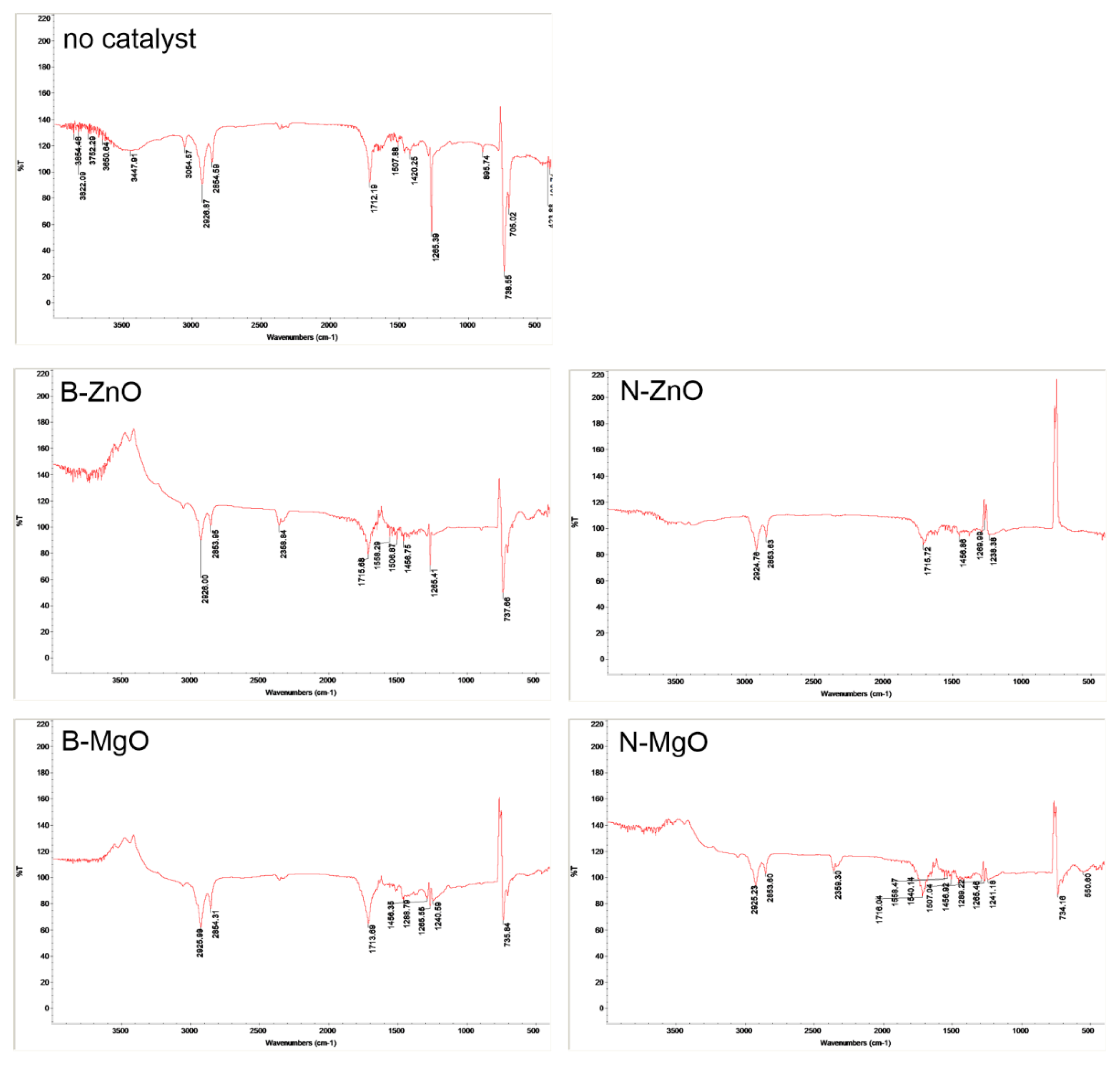

Supplement: Figure SI8 — FTIR spectrums of the pyrolytic tar at 800 °C. [file turkjchem-46-4-1306s8.tif]
